# Lateral Habenula determines long-term storage of aversive memories

**DOI:** 10.3389/fnbeh.2014.00170

**Published:** 2014-05-13

**Authors:** Micol Tomaiuolo, Carolina Gonzalez, Jorge H. Medina, Joaquin Piriz

**Affiliations:** ^1^Instituto de Biología Celular y Neurociencias, Facultad de Medicina, Universidad de Buenos AiresBuenos Aires, Argentina; ^2^Departamento de Fisiología, Facultad de Medicina, Universidad de Buenos AiresBuenos Aires, Argentina; ^3^Grupo de Neurociencia de Sistemas, Instituto de Fisiología y Biofísica Houssay (CONICET-UBA), Facultad de Medicina, Universidad de Buenos AiresBuenos Aires, Argentina; ^4^Instituto de Fisiología Biología Molecular y Neurociencias (IFIBYNE-CONICET), Universidad de Buenos AiresBuenos Aires, Argentina

**Keywords:** Lateral Habenula, memory persistence, fear memory, rats, Wistar, inhibitory avoidance learning

## Abstract

The Lateral Habenula (LHb) is a small brain structure that codifies negative motivational value and has been related to major depression. It has been shown recently that LHb activation is sufficient to induce aversive associative learning; however the key question about whether LHb activation is required for an aversive memory to be formed has not been addressed. In this article we studied the function of the LHb in memory formation using the Inhibitory Avoidance task (IA). We found that LHb inactivation during IA training does not disrupt memory when assessed 24 h after, but abolishes it 7 days later, indicating that LHb activity during memory acquisition is not necessary for memory formation, but regulates its temporal stability. These effects suggest that LHb inactivation modifies subjective perception of the training experience.

## Introduction

The Lateral Habenula (LHb) is a small epithalamic structure that exerts a powerful inhibitory control over dopaminergic brain stem centers and conveys negative motivational signals. Recently it has been shown that activation of LHb neurons (Friedman et al., 2011; Lammel et al., 2012), LHb axonal terminals (Stamatakis and Stuber, 2012) or LHb excitatory inputs (Shabel et al., 2012) is sufficient to drive conditioned place avoidance. Those results clearly indicate that pairing LHb activation with a given stimulus is sufficient to induce the formation of a conditioned aversive memory. Nevertheless, the fundamental question of what is the role of the LHb in the processes of aversive memory formation has not been clarified.

Previous works approaching that issue have relied on lesions comprising LHb and Medial Habenula or the Fasiculus Retroflexus, making their result difficult to interpret (Wilcox et al., 1986; Thornton and Bradbury, 1989; Vale-Martínez et al., 1997), or used LHb electrical stimulation and found frequency dependent subtle effects over memory acquisition (Shumake et al., 2010; Ilango et al., 2013). Besides, one caveat regarding all of those works is that they utilized behavioral protocols that involve learning across multiple trials, making it impossible to dissociate between effects of LHb manipulations on retrieval, reconsolidation and acquisition.

In this article we overcome those issues taking advantage of the step down Inhibitory Avoidance (IA) task in which animals learn not to step down from a platform in order to avoid a mild foot shock. IA is acquired in one single trial, which makes it ideal for studying processes initiated by training, uncontaminated by prior or further trials, rehearsals, or retrievals (Gold, 1986; Izquierdo and Medina, 1997).

The mesocorticolimbic dopaminergic system has been traditionally identified as the key component of reward related behavior (Schultz, 2007). In addition, recent articles have consistently shown VTA dopamine system to be sufficient and necessary for associative learning (Fadok et al., 2009; Tsai et al., 2009; Zweifel et al., 2009). In addition, to this well-known role, VTA dopaminergic transmission modulates temporal stability of memories (Rossato et al., 2009; Guzmán-Ramos et al., 2010; Kramar et al., 2014).

IA has been a fruitful paradigm to investigate the mechanisms that mediate temporal stability of long-term memory (LTM). In this paradigm, by changing the intensity of the electric shock during training, it is possible to modulate temporal stability of IA LTM. A weak shock training (0.4 mA) generates a LTM that lasts for 24–48 h while a stronger shock (0.8 mA) generates a LTM that lasts for 7 days or longer. Using the IA and other protocols it was possible to determine that temporal stability of LTMs is under the control of the mesolimbic dopaminergic system (Rossato et al., 2009; Guzmán-Ramos et al., 2010; Kramar et al., 2014).

Specifically in the IA paradigm, increasing or decreasing D1/D5 receptors activation in the hippocampus has been found to extend or reduce temporal stability of fear memories, respectively (Rossato et al., 2009; Katche et al., 2013). In addition, it has been demonstrated that different structures are involved in the process that modulates IA LTM temporal stability at different time points after acquisition. Hence, in the hippocampus temporal stability of IA LTM is modulated by dopamine 12 h after acquisition, during a late critical window for memory consolidation in which BDNF synthesis in the hippocampus induced by VTA borne dopaminergic activity, is necessary and sufficient to ensure IA LTM stability for 7 days or longer (Bekinschtein et al., 2007; Rossato et al., 2009). The 12 h window seems to be a critical period for memory consolidation which is also present in other brain structures like the restrospenial cortex (Katche et al., 2013) and the amygdala (Ou et al., 2010).

In addition, specifically during this lapse, stressful events have a major impact over temporal stability of the previously acquired memory (Yang et al., 2013). On the other hand, dopaminergic activity in mPFC seems to modulate IA LTM temporal stability earlier, at the moment of the acquisition (Lauzon et al., 2012; Gonzalez et al., unpublished results).

Here we studied the role of the LHb in the process of formation and maintenance of IA memory. We found that inactivation of the LHb during training selectively impairs the temporal stability of the IA LTM memory, leaving its formation intact. We found this impairment to be reversed by several manipulations that increase temporal stability of the IA memory formed by weak shock training, standing out a similarity between this memory and the memory formed during LHb inactivation. Consequently, our results indicate that LHb activity is not necessary for the formation of an aversive memory, but ensures its temporal maintenance. In addition, our results suggest that during aversive associative learning activity of the LHb modulates the subjective negative value attributed to the experience.

## Materials and methods

### Subjects

Experiments were conducted in male Wistar rats from the vivarium of the Italian Hospital (Buenos Aires, Argentina) weighting 230–260 g. Animals were housed five to a cage and kept at a constant temperature of 22°C, with water and food *ad libitum*, under a 12-h light/dark cycle (lights on at 8:00 am). Each animal was used only for one experiment. Experimental procedures followed the guidelines of the USA National Institutes of Health Guide for the Care and Use of Laboratory Animals and were approved by the Animal Care and Use Committees of the University of Buenos Aires (CICUAL).

### Surgery

Rats were bilaterally implanted under deep ketamine/xylacine anesthesia (100 and 5 mg/kg, respectively) with 22-g guide cannulae aimed to the LHb (AP −3.3 mm, LL ± 0.7 mm, DV −3.8 mm), mPFC (AP +3.20 mm, LL ± 0.75 mm, DV −3.20 mm) or dorsal CA1 region of the hippocampus (AP −3.9 mm, LL ± 3.0 mm, DV −1.4 mm) (from Bregma). Coordinates were based on Paxinos and Watson (2007). Cannulae were fixed to the skull with dental acrylic. At the end of surgery, animals were injected with a single dose of meloxicam (0.2 mg/kg) as analgesic and gentamicin (2.5 mg/Kg) as antibiotic. Behavioral procedures commenced 5–7 days after surgery.

### Inhibitory avoidance training and testing

After recovery from surgery, animals were handled once a day for 2 days and then trained in the IA as described previously (Bekinschtein et al., 2007). During the handling session animals were manipulated in the same form they were during intracerebral infusions. Briefly, they were grasped by hand and slightly restrained in the lap or the arm of the investigator. During the second day of this manipulation in most animals there were no evident signs of stress. For training, animals were gently placed on the platform and, as they stepped down onto the grid, received a single 3-s, 0.8 mA scrambled foot shock or a 3-s, 0.4 mA scrambled foot-shock (weak training). The parameter evaluated during training and testing sessions is the latency to step down from the platform. Rats were tested for retention either at 24 h or 7 days after training. In the test sessions the foot shock was omitted. All animals were tested only once. Training was always performed between 10 and 11 am.

### Drug infusions

For intracerebral infusions, 30-Gauge needles connected to Hamilton syringes were used. The volume infused was 0.5 μ l/side (LHb) and 1 μ l/side (mPFC and hippocampus) and the infusion rate was 0.25-0.5 μ l/min. Infusions were delivered through a needle extending 1 mm beyond the tip of the guide cannula. The needle was left in place for additional 120 s to minimize backflow. During the procedure, the animals were slightly restrained with the hands, without provoking any evident stress as mentioned in the previous section. Drugs and doses were as follow: anisomycin, 50 μg/side; emetine, 50 μg/side; muscimol, 30 ng/side; SKF 38393, 12.5 μg/side; human-BDNF (referred as BDNF), 0.5 μg/side. Drugs were dissolved in saline, except for SKF 38393 (saline 10% DMSO). Drugs were purchased from Sigma-Aldrich, except BDNF, purchased from Alomone labs.

### Histology

Cannula placement was checked after the end of the behavioral procedures. Animals were killed and the brains were extracted, fixed in 4% PFA for 48 h and sliced in 100 μm coronal sections. Cannula placement was figured out based on the lesion made by the injector cannula. Only data from animals with cannulae located in the intended site were included in the final analysis. To corroborate that injection site could be inferred as mentioned, in few animals we injected equivalent volume of Rhodamine labeled α-Bungarotoxin (0.5 μg/μl) and checked localization by the fluorescence (Figure S1).

### Open field and elevated plus maze tests

To evaluate locomotor activity, animals were exposed to an open field. The open field is a 50 cm high, 50 cm wide, and 39 cm deep arena with black plywood walls and a brown floor divided into nine squares by black lines. The number of line crossings was measured manually during each minute, in a 5 min test session. To evaluate anxiety state, animals were exposed to an elevated plus maze. The maze is a cross-shaped maze with two open arms and two closed arms, which is elevated 100 cm above the floor. The arms are 50 cm wide, 10 cm depth and the panels of the closed arms have a 45 cm high. The total number of entries into the closed and open arms and the time spent in the open arms were recorded over a 5 min session.

### Data analysis

IA experiments were analyzed by repeated measures ANOVA using the treatment (drug infused x time of testing) as the unpaired factor and training and testing step down latencies as the paired factor (named training in the text). ANOVA was followed by Bonferroni multiple comparison test. Degrees of freedom of the training factor were always one since we compared step down latencies before and after training, therefore this value is omitted in the text. In addition, training effect was always found significant; thus for the sake of simplicity we omitted “*F*” values associated with this effect in the text. All other experiments were analyzed by unpaired Student's *t*-test. For the sake of visual simplicity symbols showing significant differences between step-down latencies of training and testing were omitted. Data in the bar graphs are presented as mean ± SEM. Numbers on front of the bar graphs represents the number of animals per group.

## Results

**Figure 1:**
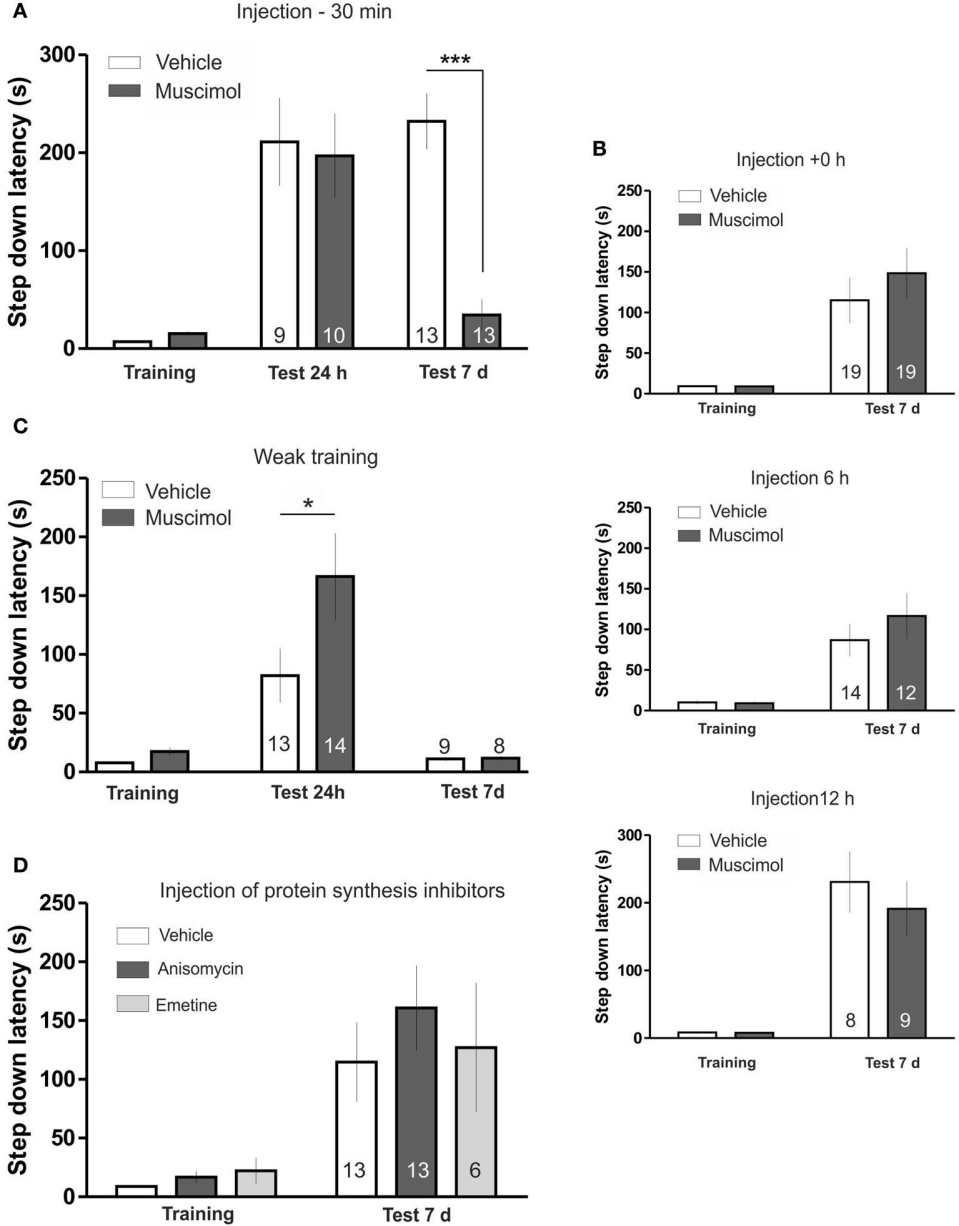
**(A)** LHb inactivation disrupts temporal stability of IA memory. Animals were trained in the IA 30 min after injection of muscimol or vehicle (saline) in the LHb. Muscimol injection did not affect memory retention in the 24 h tested group but induced a clear memory impairment in the 7 days tested group. **(B)** Top: Injection of muscimol immediately after IA training did not induce amnesia. Centre: Injection of muscimol 6 h after IA training did not induce amnesia. Bottom: Injection of muscimol 12 h after IA training did not induce amnesia. **(C)** Temporal stability of weak IA LTM is not impaired by LHb inactivation. Rats were trained in the IA using a weak training 30 min after injection of muscimol or vehicle in the LHb. Muscimol injection facilitated memory tested 24 h after training but did not affect retention 7 days after training. **(D)** Inhibition of protein synthesis in the LHb during training does not disturb temporal stability of IA LTM. Injection of anisomycin or emetine in the LHb 15 min before IA training did not impair memory retention. ^*^p < 0.05; ^***^p < 0.001.

To determine if the LHb is involved in the maintenance of a fear-motivated memory, we trained rats in the IA task 30 min after the bilateral injection of the GABAergic agonist, muscimol in the LHb. Using this drug we can selectively inhibit the LHb, since the Medial Habenula does not present GABA-A receptor signaling (Wang et al., 2006). Test session was carried out 24 h or 7 days later. The results are summarized in Figure 1A. LHb inactivation did not affect memory evaluated 24 h after training; however it induced a marked amnesia when memory was assessed 7 days after training [ANOVA_(3, 40)_; *F*_(treatment)_ = 7.516, *p* < 0.0001; *F*_(interaction)_ = 9.196, *p* < 0.0001; *post hoc* comparisons: test 24 h: Vehicle vs. Muscimol, ns; test 7 days: Vehicle vs. Muscimol ^***^p < 0.001; training Muscimol vs. test 7 days Muscimol, ns]. These findings indicate that blocking LHb activity during acquisition specifically affects IA memory maintenance, leaving memory formation intact.

Shock perception was not grossly disturbed in muscimol-infused animals since we observed no differences in the time animals spent before returning to the platform after the beginning of the shock both in weak and strong training (Figures S2A,B). On the other hand, we observed a tendency toward an increased step-down latency during training in the muscimol injected group (Vehicle: 7.24 ± 1.03, *N* = 22; Muscimol: 15.38 ± 1.92, *N* = 22, *p* > 0.05, Bonferroni *post hoc* test after Two-Way ANOVA) that was not correlated with memory performance at day 7th during the test session (Pearson *r* = −0.1413, *p* = 0.6452) and was also observed with other manipulations that did not impair memory persistence (see Figure 1D). Therefore, deficits in LTM maintenance induced by LHb inhibition cannot be explained by an altered training performance or sensitivity to an electric shock.

To control for general effects of LHb blocking over motility or anxiety we analyzed the effect of the infusion of muscimol into the LHb 30 min before subjecting animals to an open field and an elevated plus maze tests. The inhibition of LHb neuronal activity did not affect open field exploratory activity (Figure S2C), the number of entries into closed or open arms of the elevated plus maze, or the time spent in the open arms (Figure S2D). Thus, the impairment in memory persistence caused by LHb inactivation cannot be attributed to general effects over animals' motility or anxiety state.

We further tested specificity of the pre-training LHb blockade effect by injecting muscimol 1 mm lateral to the LHb (1.7 mm lateral). This manipulation did not alter memory performance 7 days after training (Figure S2E). Since structures dorsal to the LHb like the ventricle and the Dentate Gyrus extended laterally up to this coordinates, this experiment also ruled out that the behavioral effect observed after LHb inactivation could be attributed to unspecific inactivation of the Dentate Gyrus or to spillover of muscimol into the ventricle.

To investigate the time window during which LHb activity is involved in modulating temporal stability of the IA LTM, we inactivated the LHb immediately after training, 6 or 12 h later (Figure 1B). Statistical analyses revealed that none of those manipulations modified memory performance evaluated 7 days after training indicating that LHb activity is involved in determining temporal stability of IA LTM during a restricted time window [+0 h: ANOVA_(1, 36)_; *F*_(treatment)_ = 0.65, *p* = 0.4261; *F*_(interaction)_ = 0.66; 6 h: ANOVA_(1, 24)_; *F*_(treatment)_ = 0.6224, *p* = 0.4379; *F*_(interaction)_ = 0.9629, *p* = 0.3362; 12 h: ANOVA_(1, 15)_; *F*_(treatment)_ = 0.44, *p* = 0.5181; *F*_(interaction)_ = 0.43, *p* = 0.5206].

Temporal stability of short- and long-lasting IA LTM is mediated by two different processes that decay at different rates (Bekinschtein et al., 2007; Rossato et al., 2009). We analyzed if LHb inactivation had an effect over temporal stability of memories regardless of their strength. For this, we infused muscimol in the LHb before weak IA training (see Materials and Methods). Using this protocol a short-lasting IA LTM is formed, which lasts for 24 h, but not for 7 days (Figure 1C; Vehicle: training vs. test 24 h, *p* < 0.05; training vs., test 7 days, ns). Temporal stability of this non-persistent memory was not impaired by LHb inactivation. In fact we found significant memory facilitation in muscimol infused group 24 after training (Figure 1C) that was not evident 7 days after training [ANOVA_(3, 40)_; *F*_(treatment)_ = 6.928, *p* < 0.001; *F*_(interaction)_ = 7.817, *p* < 0.001; *post hoc* comparisons: test 24 h, Vehicle vs. Muscimol, ^*^p < 0.01; test 7 days, Vehicle vs. Muscimol, ns]. Overall, this experiment indicates that LHb inactivation selectively affects temporal stability of stable IA memory.

Having found that LHb inactivation during acquisition disrupt temporal stability of IA memory we wondered whether that process depends also on LHb *de novo* protein synthesis. For that purpose, we injected the protein synthesis inhibitors anisomycin or emetine 15 min before training (Figure 1D); a time window where they have been shown to effectively block protein synthesis in other structures (Bekinschtein et al., 2007; Gold, 2008; Katche et al., 2013). Statistical analysis indicated that neither of these two compounds affected memory persistence [ANOVA_(2, 28)_; *F*_(treatment)_ = 0.4474, *p* = 0.6438; *F*_(interaction)_ = 0.4685, *p* = 0.6308), indicating that training induced protein synthesis in the LHb is not necessary for ensuring temporal stability of IA memory.

Our results indicate that IA LTM formed by a strong training under LHb inactivation lasted for 24 h but not 7 days, a profile similar to the IA memory formed by weak training (Bekinschtein et al., 2007; Rossato et al., 2009; Katche et al., 2013). We were intrigued by this parallelism and decided to analyze the similarities between those two conditions. For this purpose, we investigated if manipulations that are known to increase temporal stability of weak training IA LTM do also extend temporal stability of the IA memory formed under LHb inactivation.

Previous works have characterized a critical window for the regulation of IA LTM temporal stability that takes place 12 h after acquisition. During this period increasing D1/D5 receptors activity or BDNF signaling in the hippocampus extend temporal stability of weak IA LTM making it stable for more than 1 week (Bekinschtein et al., 2007; Rossato et al., 2009). To investigate if these manipulations reverse the effect of LHb inactivation, we performed double bilateral cannulation experiments, aiming cannulae at the LHb and at the dorsal hippocampus.

**Figure 2:**
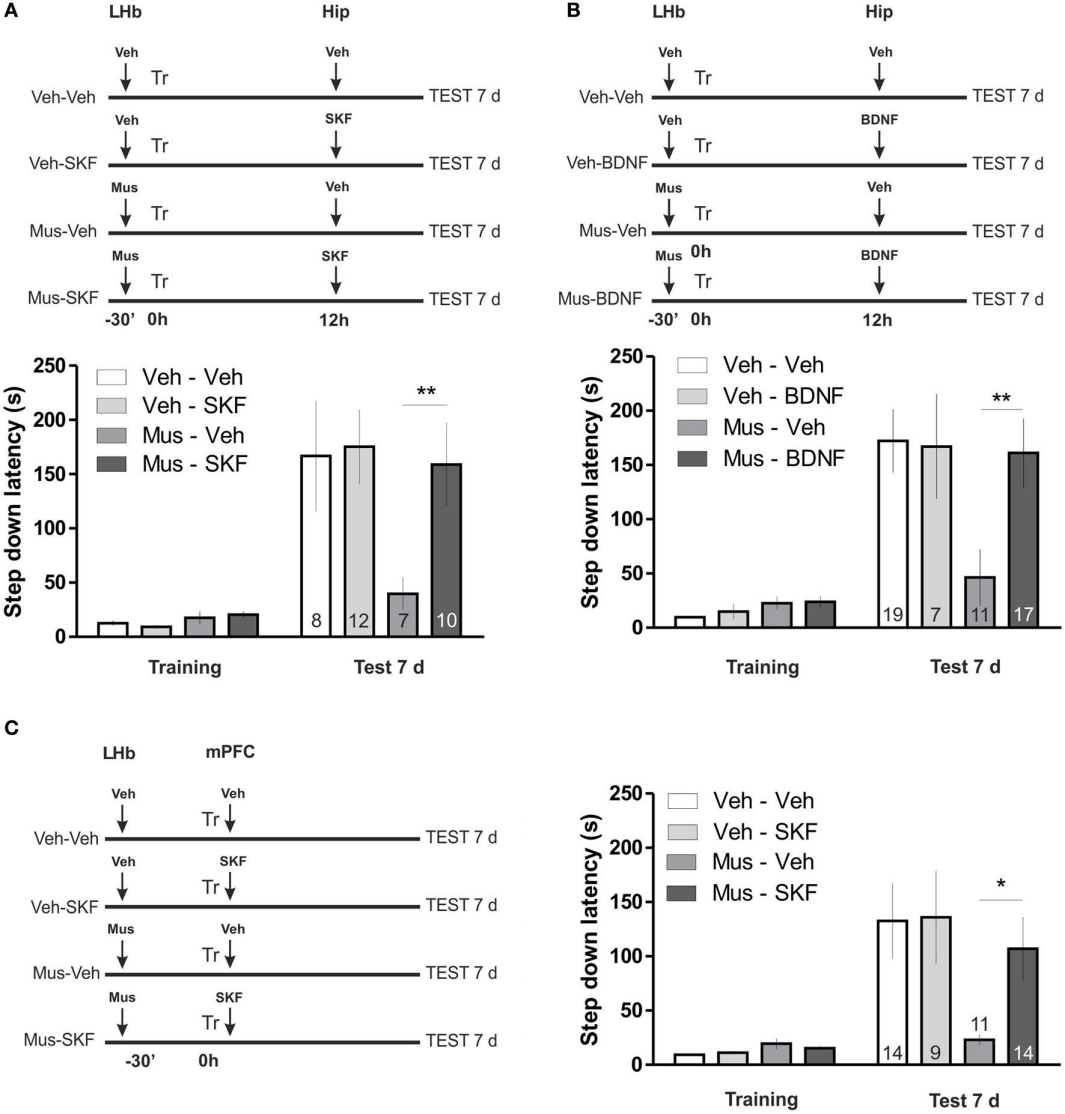
**(A)** Activation of D1/D5 receptors in the hippocampus 12 h after training reverses memory maintenance deficits induced by LHb inactivation. Top: diagram illustrating the protocol used. Bottom: hippocampal infusion of SKF-38393 12 h after training reversed memory deficits induced by inhibition of the LHb during training. **(B)** BDNF in the hippocampus 12 h after training reverses memory maintenance deficits induced by LHb inactivation. Top: diagram illustrating the protocol used. Bottom: hippocampal infusion of BDNF reversed memory deficits induced by inhibition of the LHb during training. **(C)** Activation of D1/D5 receptors agonist in the mPFC after training reverses LHb inactivation induced amnesia. Left: diagram illustrating the protocol used. Right: delivering of SKF-38393 into mPFC immediately after IA training prevented memory deficits induced by intra LHb muscimol infusion. ^*^p < 0.05; ^**^p < 0.01.

In the first experiment we inactivated the LHb before a strong IA training, as in previous experiments, and 12 h later we delivered the D1/D5 agonist SKF-38393 in the hippocampus (Figure 2A). We found this manipulation to reverse the deficit in memory maintenance induced by LHb inactivation. Indeed statistical analysis confirmed that the amnesic effect of LHb inactivation is not present in animals injected with SKF in the hippocampus 12 h later [ANOVA_(3, 33)_; *F*_(treatment)_ = 2.06, *p* = 0.1252; *F*_(interaction)_ = 2.64, *p* = 0.0654; *post hoc* comparisons: test 7 days, Vehicle—Vehicle vs. Muscimol—Vehicle, ^**^p < 0.01; test 7 days, Vehicle—Vehicle vs. Muscimol—SKF, ns; test 7 days, Muscimol—Vehicle vs. Muscimol—SKF, ^**^p < 0.01].

In the second experiment we repeated the same schema, infusing BDNF in the dorsal hippocampus 12 h after training (Figure 2B), a manipulation that was also demonstrated to increase temporal stability of weak IA memories (Bekinschtein et al., 2007). Accordingly, this manipulation also reversed the effect of LHb inactivation [ANOVA_(3, 50)_; *F*_(treatment)_ = 2.35, *p* = 0.0839; *F*_(interaction)_ = 3.54, *p* = 0.0210; *post hoc* comparisons: test 7 days, Vehicle—Vehicle vs. Muscimol—Vehicle, ^***^p < 0.001; test 7 days, Vehicle—Vehicle vs. Muscimol—BDNF, ns; test 7 days Muscimol—Vehicle vs. Muscimol—SKF, ^**^p < 0.01]. These two results suggest that similar molecular mechanisms promote temporal stability of weak IA LTM and the IA memory formed under LHb inactivation. We next wondered if a manipulation in other brain structure at a different time point which is also known to promote temporal stability of weak IA, does also counteract the effect of LHb inactivation. Recently, it has been found that D1/D5 signaling in the mPFC plays a role in IA LTM temporal stability. Particularly, infusion of D1/D5 agonist during acquisition increases temporal stability of weak IA LTM (Gonzalez et al., unpublished observation). We then performed double cannulation experiments in which we infused muscimol in the LHb, 30 min before training and D1/D5 agonist SKF-38393 in the mPFC immediately after training (Figure 2C). Notably, this manipulation did also reverse the effect of LHb inactivation [ANOVA_(3, 44)_; *F*_(treatment)_ = 2.21, *p* = 0.1005; *F*_(interaction)_ = 3.13, *p* = 0.0352; *post hoc* comparisons: test 7 days: Vehicle—Vehicle vs. Muscimol—Vehicle, ^**^p < 0.01; Vehicle—Vehicle vs. Muscimol—SKF, ns; Muscimol—Vehicle vs. Muscimol—SKF, ^*^p < 0.05]. These results indicate that the similarities between consolidation mechanisms of LTMs formed by weak training and by strong training under LHb inactivation comprise at least two structures (mPFC and hippocampus) and two different time points (0 h and 12 h after training, respectively).

## Discussion

In this article we analyzed the role of LHb activity during acquisition of the IA, a single trial paradigm of aversive learning. Our main finding is that LHb activity during training is not necessary for IA LTM formation, but is critical to ensure its temporal stability.

Since Hikosaka's seminal work (Matsumoto and Hikosaka, 2007), accumulating evidences indicate that the LHb exerts a crucial role in coding negative reward prediction error, acting on an opposite direction to reward activated VTA dopaminergic neurons (Hikosaka, 2010). Hence, activation of the LHb by a given experience would say that things went worse than expected. Thus, hereafter the sequences of actions and the context associated with that experience will be avoided. This idea is fully supported by recent articles in which LHb activity was shown to be sufficient to induce aversive associative learning (Lammel et al., 2012; Shabel et al., 2012; Stamatakis and Stuber, 2012). However, those findings do not implicate a prediction about whether an intrinsically negative experience would need the LHb to generate an aversive associative learning.

In terms of classical negative associative learning, a negative experience could be decomposed into an intrinsically aversive unconditioned stimulus and a group of contingent neutral stimuli which will be avoided following the pairing with the unconditioned stimulus. If the LHb would be necessary for the experience to be perceived as negative, LHb inactivation would disrupt the aversive associative learning induced by the unconditioned stimulus presentation. On the other hand, an effect of LHb inactivation over memory formation could also indicate that the LHb is related to memory storage functions.

Our results contradict both ideas since we found that LHb inactivation or protein synthesis inhibition does not disrupt IA learning. Instead, we found a marked effect of LHb inactivation over IA LTM temporal stability, indicating that the LHb plays a complex role in associative learning which is related to the processes that modulate LTM temporal stability.

Previous works analyzing the role of the LHb in memory processes have used behavioral protocols that involve several trials which might include cofounding effects of the LHb during retrieval and/or reconsolidation. They have shown that Habenula lesions disrupt acquisition of the Morris Water Maze (Lecourtier et al., 2004), or that pharmacological inactivation of the LHb disrupts retrieval of the object recognition test (Goutagny et al., 2013). Other set of papers analyzed the role of the LHb in the formation of aversive memories using the active avoidance paradigm (Wilcox et al., 1986; Thornton and Bradbury, 1989; Vale-Martínez et al., 1997). Those works have relied on lesions of the LHb and adjacent structures like the Medial Habenula or the Fasciculus Retroflexus making their results difficult to interpret. They reported disparate results ranging from no effect (Vale-Martínez et al., 1997), effects attributed to Medial Habenula lesion (Wilcox et al., 1986) or described effects dependent on training intensity (Thornton and Bradbury, 1989). In addition, electrical stimulation of the LHb, using a presumably excitatory protocol, has been found to disrupt the acquisition of the two way active avoidance task (Shumake et al., 2010; Ilango et al., 2013). Therefore, a clear picture about how the LHb is involved in memory formation processes has not emerged so far. To the best of our knowledge this is the first study analyzing the effect of LHb inactivation on the acquisition of a single trial aversive memory paradigm.

As mentioned in the introduction, how long the IA memory persists is primarily defined by the intensity of the foot shock during training (Bekinschtein et al., 2007; Rossato et al., 2009; Katche et al., 2010). Notably, LHb inactivation during weak shock training did not disturb this temporal profile. In fact, 24 h after training with a weak shock, we found a significant increase in the step down latency of muscimol infused animals, compared with vehicle animals. The interpretation of this observation is not straightforward, since both groups showed significant memory, making it difficult to discriminate between a stronger fear memory and a change in the step down latency associated with decision making processes (see Stopper and Floresco, 2014). Nevertheless, our results strongly suggest that inhibition of the LHb during acquisition does not exert a general effect over temporal stability of IA LTM, but selectively disrupt temporal stability of memories that are meant to be conserved for long periods of time.

We found that LHb inactivation only disrupts IA LTM temporal stability when exerted before training, indicating that LHb activity is required during a time window circumscribed to the time of training. In addition, we found that inhibition of protein synthesis in the LHb does not affect IA LTM temporal stability. Since brain regions responsible of memory storage and retrieval undergo protein synthesis dependent plastic changes after the acquisition of new information, this result suggest that the LHb is not involved in the storage of the newly formed memory (Alberini, 2008). Therefore, those results delineate a picture in which the LHb is not involved in IA LTM storage but its activity at the moment of training is required to generate a signal that boost temporal stability of the IA memory.

What is the nature and meaning of that signal? The fact that temporal persistence of the IA memory is primarily determined by shock intensity during training suggests the magnitude of the negative experience determines how long the memory would persist. We found that LTM IA memory formed under LHb inactivation lasted for 24 h but not 7 d, similarly to the IA LTM formed by weak shock training. We then challenged this correlation by three manipulations (see Figure 2) that are known to increase temporal stability of weak shock training IA LTM and that involved different brain structures, time points and biochemical pathways, namely infusion of D1/D5 agonist in the mPFc before training (Gonzalez et al., unpublished results) or in the hippocampus 12 h after training (Rossato et al., 2009) or BDNF infusion in the hippocampus 12 h after training (Bekinschtein et al., 2007). We found that all of them were able to reverse the effect of LHb inactivation. Hence those striking results suggest that somehow LHb inactivation renders a strong IA training a weak one.

The easiest explanation for that observation would be that LHb inactivation reduces shock sensitivity; however this explanation is very unlikely since we found no differences between the time vehicle and muscimol groups received the shock before returning to the platform and we observed that muscimol infused animals acquired a 24 h lasting IA LTM following a weak training (Moncada and Viola, 2007). Alternatively it could be hypothesized that the LHb modulates the subjective aversive value of the IA training experience. Hence, LHb inactivation during training would not block IA learning but instead would reduce the subjective aversive value attributed to the experience, reducing the strength of the memory associated with it. The attribution of this role to the LHb is widely supported by physiological data showing that LHb neurons encodes negative reward value (Matsumoto and Hikosaka, 2007), that pairing of a context with LHb activation is enough to generate conditioning place avoidance (Lammel et al., 2012; Shabel et al., 2012; Stamatakis and Stuber, 2012), and with a recent paper demonstrating that the LHb modulates subjective reward preferences (Stopper and Floresco, 2014). On the other hand, weak training IA LTM seems to be independent of the activity of the LHb, highlighting the existence of different mechanisms behind LTM formation and temporal maintenance, which might depend differently on the subjective relevance attributed to aversive experiences.

A second question that arises is how the LHb relates to other structures manipulated here (the hippocampus and mPFC) and how their interaction modulates LTM temporal stability. The effect of LHb inactivation is reversed by injection of D1/D5 agonist in the mPFC, a striking observation since most evidences indicate that the LHb inhibits dopaminergic activity (Christoph et al., 1986; Ji and Shepard, 2007; Matsumoto and Hikosaka, 2007). However, this apparent contradictory observation can be explained taking into account the work of Lammel et al. (2012), who have recently characterized a subpopulation of VTA dopaminergic neurons that are excited by the LHb and project to mPFC. Furthermore, the same authors have shown that this LHb-VTA-mPFC circuit is involved in encoding aversive learning. Therefore, it is plausible to postulate that activation of the LHb during training increases dopaminergic release in the mPFC through this newly described pathway. In turns, dopaminergic signaling in that structure would be necessary for temporal stability of IA memory (Gonzalez et al., unpublished observations).

On the other hand, how the LHb and the hippocampus interact in the processes that modulate temporal stability is not evident from our results. Two recent works have described that dorsal hippocampus and the LHb are synchronized in the theta frequency range and that such interaction might modulate memory recall (Aizawa et al., 2013; Goutagny et al., 2013). However, we manipulated hippocampus 12 h after training, a moment at which we found LHb inactivation does not disturb LTM. Therefore, a parsimonious explanation would be that the processes that modulate LTM temporal stability are triggered during the acquisition and require LHb activity; then consolidation takes place during several hours and the temporal stability of the LTM is defined in, at least, one critical window that starts around 12 h after acquisition, requires dopaminergic and BDNF signaling in the hippocampus and might not be related to LHb activity.

In conclusion our results demonstrate a novel role for of the LHb in aversive associative learning. This role is complex and is not related to coding of the aversive experience or LTM storage, instead the LHb seems to modulate the strength of the aversive experience. Therefore, the LHb might play a general role in the processing of aversive or fearful experiences. To understand such a role might be relevant for trauma related syndromes like post-traumatic stress disorders.

### Conflict of interest statement

The authors declare that the research was conducted in the absence of any commercial or financial relationships that could be construed as a potential conflict of interest.

## Supplementary material

Figure S1 **(A–C)** Photomicrographs illustrating the lesion caused by the cannula; the “X” indicates the place that correspond to the area of drug infusion, in LHb **(A)**, hippocampus **(B)**, and mPFC **(C)**. **(D–F)** Photomicrographs illustrating the fluorescence observed after infusion of rhodamine labeled α-Bungarotoxin in the LHb **(D)**, hippocampus **(E)**, and mPFC **(F)** in brains of rats that were previously used for experiments. Pictures are merged images of transmitted light and red fluorescence pictures. Volumes of rhodamine labeled α-Bungarotoxin and infusion procedures were the same used for drug infusion during experiments. Scale bar: 1 mm.

Figure S2 **(A,B)** Bar diagrams illustrate the time animals received the shock before returning to the platform during a strong **(A)** or weak training **(B)**; no differences were detected (Student's *t*-test: *p* = 0.6373 and *p* = 0.9259 for strong and weak shock, respectively). **(C)** Bar graph represents the number of quadrant crosses in a novel open field. Exploratory activity is not modified by muscimol injection in the LHb (Student's *t*-test, *p* = 0.99). **(D)** Elevated plus maze performance is not modified by muscimol injection in the LHb. Left: number of entries into closed (Student's *t*-test, *p* = 0.3297,) and open arms of the maze (Student's *t*-test, *p* = 0.8581); right: time spent in the open arms (Student's *t*-test, *p* = 0.1350).**(E)** pre-training injection of muscimol 1 mm lateral to the LHb (1.7 mm lateral from bregma) did not induce IA amnesia [ANOVA_(1, 13)_; *F*_(treatment)_ = 0.0235, *p* = 0.8805; *F*_(interaction)_ = 0.074, *p* = 0.7898; *post hoc* comparisons: test 7d, Vehicle vs. Muscimol, ns].
