# Supplementary figures and images for: Lateral Habenula determines long-term storage of aversive memories
Source: Front Behav Neurosci. 2014 May 13;8:170. doi: 10.3389/fnbeh.2014.00170 (PMC4026688; doi:10.3389/fnbeh.2014.00170)

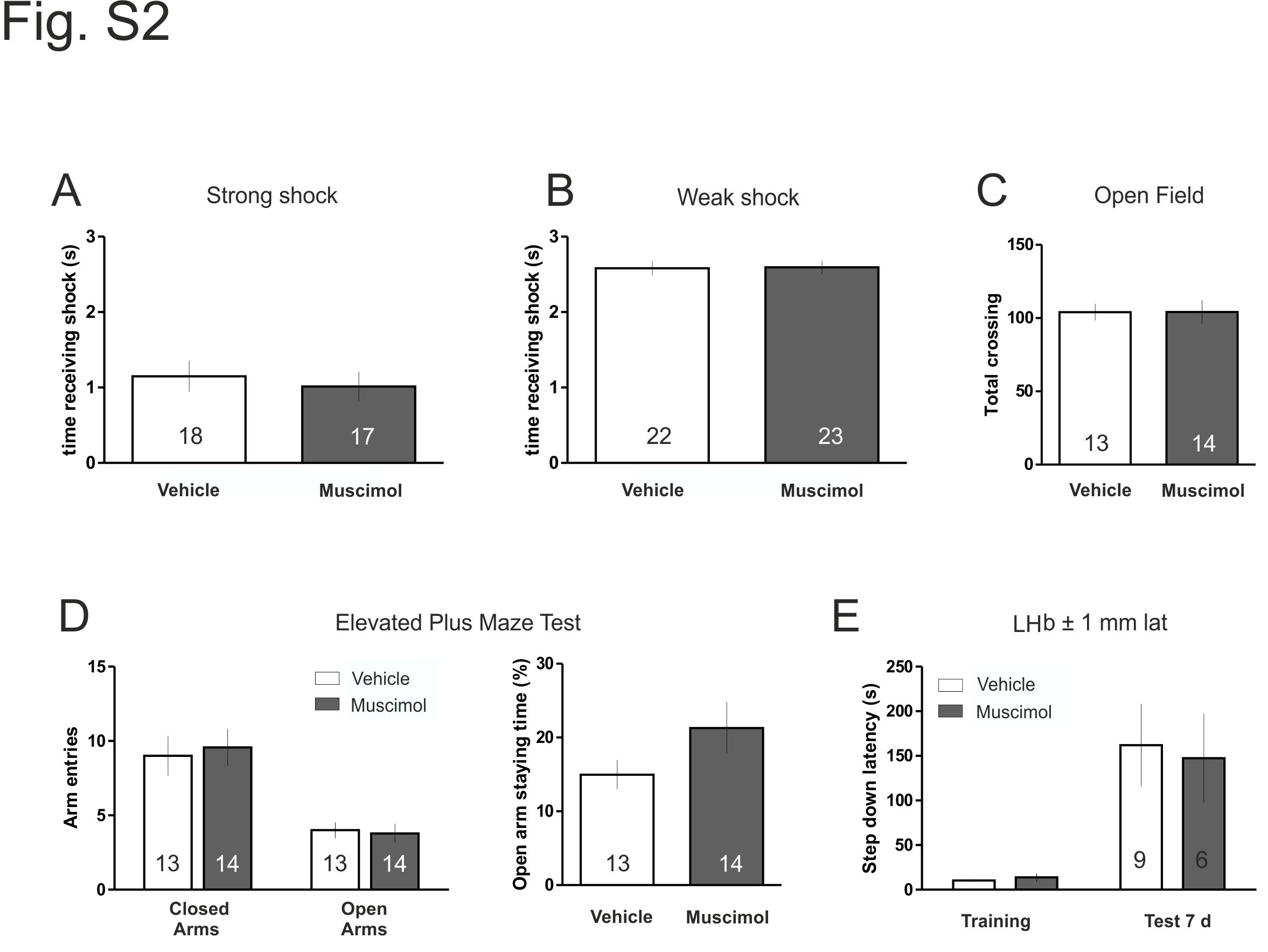

Supplement: Figure S1 — (A–C) Photomicrographs illustrating the lesion caused by the cannula; the “X” indicates the place that correspond to the area of drug infusion, in LHb (A), hippocampus (B), and mPFC (C). (D–F) Photomicrographs illustrating the fluorescence observed after infusion of rhodamine labeled α-Bungarotoxin in the LHb (D), hippocampus (E), and mPFC (F) in brains of rats that were previously used for experiments. Pictures are merged images of transmitted light and red fluorescence pictures. Volumes of rhodamine labeled α-Bungarotoxin and infusion procedures were the same used for drug infusion during experiments. Scale bar: 1 mm. [file Presentation1.ZIP › Piriz_Supplementary_Figure_1.JPEG]

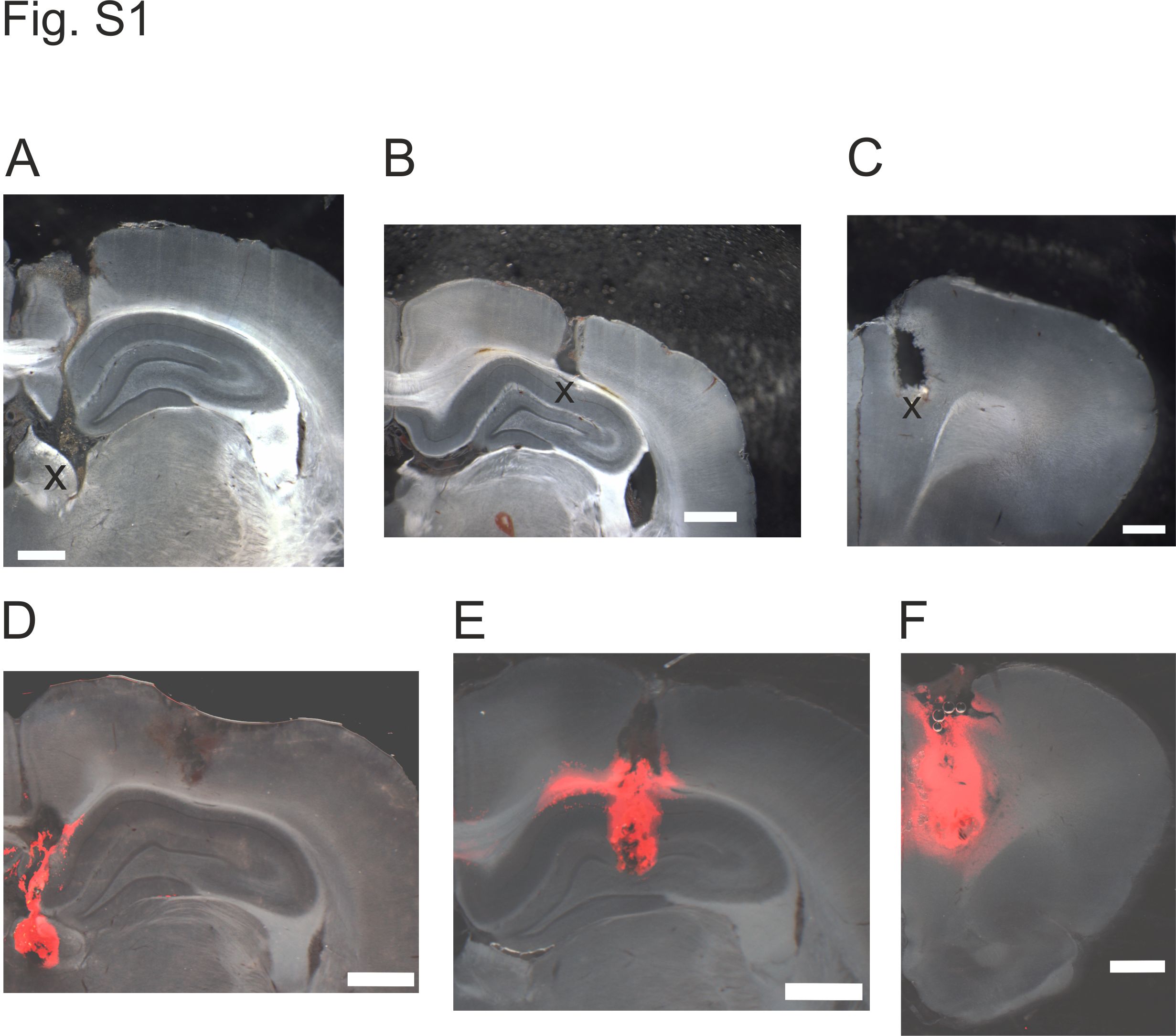

Supplement: Figure S1 — (A–C) Photomicrographs illustrating the lesion caused by the cannula; the “X” indicates the place that correspond to the area of drug infusion, in LHb (A), hippocampus (B), and mPFC (C). (D–F) Photomicrographs illustrating the fluorescence observed after infusion of rhodamine labeled α-Bungarotoxin in the LHb (D), hippocampus (E), and mPFC (F) in brains of rats that were previously used for experiments. Pictures are merged images of transmitted light and red fluorescence pictures. Volumes of rhodamine labeled α-Bungarotoxin and infusion procedures were the same used for drug infusion during experiments. Scale bar: 1 mm. [file Presentation1.ZIP › Piriz_Supplementary_Figure_2.JPEG]
